# Incidence and time trends of sarcoma (2000–2013): results from the French network of cancer registries (FRANCIM)

**DOI:** 10.1186/s12885-020-6683-0

**Published:** 2020-03-06

**Authors:** Brice Amadeo, Nicolas Penel, Jean-Michel Coindre, Isabelle Ray-Coquard, Karine Ligier, Patricia Delafosse, Anne-Marie Bouvier, Sandrine Plouvier, Justine Gallet, Aude Lacourt, Gaëlle Coureau, Alain Monnereau, Simone Mathoulin-Pélissier, Emmanuel Desandes

**Affiliations:** 1 Univ. Bordeaux, Inserm, Bordeaux Population Health Research Center, Epicene team, UMR 1219, F-33000 Bordeaux, France; 2 Registre des cancers de la Gironde, Univ. Bordeaux, Inserm CIC1401, F-33000 Bordeaux, France; 3 French Network of Cancer Registries, F-31000 Toulouse, France; 4 Univ. Lille, F-59000 Lille, France; 5 Medical Oncology Department, Centre Oscar Lambret, F-59000 Lille, France; 6 Department of Biopathology, Institute Bergonié, Comprehensive Cancer Center, F-33000 Bordeaux, France; 7 Department of Medical Oncology, Centre Leon Berard, F69000 Lyon, France; 8 University Claude Bernard Lyon 1, Lyon, France; 9 Registre Général des Cancers de Lille et de sa Région, C2RC, F59000 Lille, France; 10 Isère Cancer Registry, CHU Grenoble-Alpes, F-38000 Grenoble, France; 11 Dijon University Hospital, University of Bourgogne Franche-Comté, Besançon, France; 12 Digestive Cancer Registry of Burgundy, LNC UMR1231 EPICAD, F-21000 Dijon, France; 13 Medical Information Service, Public Health Department, CHU Bordeaux, F-33000 Bordeaux, France; 14 Gironde registry of haematological malignancies, Institut Bergonié, F-33000 Bordeaux, France; 15 Clinical and Epidemiological Research Unit, INSERM CIC1401, Institut Bergonié, Comprehensive Cancer Center, F-33000 Bordeaux, France; 16 Registre National des Tumeurs Solides de l’Enfant, CHU Nancy, F-54500 Vandœuvre-lès-Nancy, France; 17 Centre de Recherche en Epidémiologie et en Statistique, EPICEA team, Université Paris-Descartes, Inserm, UMR 1153, F-75014 Paris, France

**Keywords:** Sarcoma, Incidence, Trends in incidence, France, Cancer registry

## Abstract

**Background:**

The exhaustive collection of new sarcoma cases and their second histologic review offer a unique opportunity to study their incidence and time trends in France according to the major subtypes.

**Methods:**

Data were collected from population-based cancer registries covering 22% of the French population. Crude and world age-standardized incidence rates (ASR) were estimated according to anatomic, histological and genetic groups, age and sex over the 2010–2013 period.

**Results:**

Time trends in incidence were calculated by the annual percent change over the 2000–2013 period. During the most recent period (2010–2013), 3942 patients with sarcoma were included. The ASR of soft-tissue and bone sarcomas, and gastro-intestinal stromal tumors (GIST) were 2.1, 1.0 and 0.6, respectively. For the four most frequent histological subtypes (unclassified, leiomyosarcoma, GIST and liposarcoma), the ASR ranged from 0.4 to 0.7. ASRs were 1.9 for complex genomic and 1.3 for recurrent translocation sarcomas. The time-trend analysis showed a significant increase of sarcoma incidence rate between 2000 and 2005, which stabilized thereafter. Incidence rates increased for four histological subtypes (GIST, chondrosarcoma, myxofibrosarcoma, solitary fibrous tumors) and decreased for three (leiomyosarcomas, Kaposi sarcoma and fibrosarcoma).

**Conclusion:**

To our knowledge, this study is the first to investigate sarcoma incidence based on a systematic pathological review of these cancers and on the updated sarcoma classifications. Due to the paucity of literature on sarcomas, future studies using data from population-based cancer registries should consider a standardized inclusion criterion presented in our study to better describe and compare data between countries.

## Background

Sarcomas are a heterogeneous group of rare malignant tumors derived from primitive mesenchymal cells. These tumors arise from muscle, connective tissue, supportive tissue and vascular tissue, and more than 80 histologic subtypes are included in the 2013 World Health Organization (WHO) Classification of Tumors of Soft Tissue and Bone [1]. In addition to having a multiple and complex histology, these tumors can occur in almost any anatomic site. In spite of these facts, sarcomas account for less than 1% of all adult cancers and for about 20% of all malignant solid tumors in children, adolescents and young adults [2].

From an epidemiological point of view, the lack of a unified method of reporting sarcomas has led to considerable variations in the reported incidence and time trends Sarcomas are sometimes mistaken for carcinomas of the same organ, and can involve a variety of localizations. As a consequence, 30 % of sarcomas are misclassified at initial diagnosis [3]. In addition, sarcomas encompass a wide variety of histological and molecular subtypes and are categorized in rapidly evolving phenotypic and molecular subgroup classification schemas now used for sarcoma diagnosis, which has a growing impact on the management of patients [4]. Furthermore, innovation in immune-histochemistry and molecular biology techniques in the last three decades has led to major changes in the diagnosis and classification of sarcoma subtypes.

Currently, data for sarcomas in the French population are provided by the reference networks for sarcomas that collect and manage cases of soft tissue, bone and visceral sarcomas. Reference networks propose a systematic second histologic review by expert pathologists [5–7]. A few French studies carried out by these reference networks provided world age-standardized incidence rates of 4.8 and 3.3 per 100,000 inhabitants per year for all sarcomas and soft-tissue sarcomas (STS) respectively [8, 9]. However, data from these reference networks based on the voluntary participation are not totally exhaustive.

Besides reference networks, cancer surveillance information is coming from the French Network of population-based cancer registries that exhaustively collects all newly diagnosed and confirmed cancer cases within geographical areas in France [10]. The exhaustive collection of sarcoma cases from population-based cancer registries and the systematic second review of diagnosis from reference centers offer an optimal framework to study the incidence and time trends of sarcomas in France. The incidence trends have never been studied in France and the results from other countries are divergent [11]. We undertook this study to describe sarcoma entity according to anatomic sites, histologic subtypes and genetic groups based on guidelines developed by sarcoma specialists.

## Methods

### Data sources

Cases included in this study were children and adults with sarcoma diagnosed between January 1, 2000 and December 31, 2013, and living in one of the administrative areas covered by a population-based cancer registry of the French Network (details in online supplementary material). The French sarcoma pathological reference network (RRePS) and the French reference Network for bone sarcoma and rare bone tumors (RESOS) propose a systematic second histologic review and confirmation for all diagnoses of sarcomas across France [6].

### Data collection and classification

The following data were collected for each case: general demographic characteristics of the patients (age, sex, and residence area), the date of diagnosis, the anatomical site, and the histology of the tumor according to the International Classification of Diseases for Oncology, third edition (ICD-O-3) (12).

This study included intermediate (only with a “/3” behavior) and malignant sarcomas presenting morphologic criteria described in the 2013 WHO Classification of Tumors of Soft Tissue and Bone (fourth edition), regardless of the anatomic site [1]. This recent classification includes histologic updates not defined in ICD-O-3 and new terms, synonyms, morphology and behavior codes. For this reason, and whenever possible, cases were reclassified according to the updated version. The alignments from ICD-O-3 to the 2013 WHO standard classification of tumors have been validated by a panel of sarcoma specialists (clinical and pathological experts) from sarcoma Networks (NP, JMC and IRC).

Certain alignments could not be performed: ten morphological terms not described in this updated classification (e.g. sarcoma NOS, periosteal fibrosarcoma, fascial fibrosarcoma …) have been maintained for analyses. Conversely, well differentiated liposarcoma and chondroblastoma have been changed from malignant to borderline diseases. In the same way, behaviors for dermatofibrosarcoma protuberans and pigmented dermatofibrosarcoma protuberans have been also changed from malignant to borderline with henceforth, only fibrosarcomatous dermatofibrosarcoma protuberans which is coded as malignant behavior. In our analyses, we have made the choice to keep all dermatofibrosarcomas. Indeed, we do not have the possibility to differentiate if this is a dermatofibrosarcoma borderline or malignant. Besides, endometrial stromal sarcoma NOS (89303), low grade endometrial stromal sarcoma (89313) and stromal sarcoma (89353) not described in the WHO 2013 have been also included. Additional details on the list and choice of classification systems are provided in the online supporting material (see Additional File 1).

This classification also provides new genetic and molecular data for each histologic entity allowing a better characterization of sarcomas. The same group of experts were consulted with the aim of proposing the optimal classification system for sarcomas based on the genetic profile. Two main distinct genetic groups were defined: (i) sarcomas defined with simple genetics based on recurrent translocations (e.g. Ewing sarcoma, myxoïd liposarcoma), activating or inactivating mutations (e.g epithelioid sarcoma, gastrointestinal stromal tumor), *MDM2* amplification (e.g. dedifferentiated liposarcoma, low-grade central osteosarcoma); and (ii) sarcomas with complex genomic profiles (e.g. angiosarcoma, leiomyosarcoma). Another group was defined for miscellaneous and undefined alterations. The list of histology codes according to their genetic groups is presented in the supplementary material.

This study is based on data from cancer registries gathered in the French network of cancer registries and a representative of each registry was involved in the study and approved the use of its data All French registries received an authorization to collect patient data from the data protection authority (Commission Nationale de l’Informatique et des Libertés). Ethics approval and consent to participate were not required for this study which is an observational research without direct contact with patient.

### Statistical analyses

Two datasets were used: i) the first one was used to estimate the incidence of patients diagnosed during the 2010–13 period and that included data from 19 registries; and ii) the second one was used to examine trends in the incidence from 2000 to 2013 in only 11 registries for which data were available over the entire studied period. Incidence rates were presented per 100,000 person-years.

The incidence of sarcomas was described according to 1) the anatomic group (i.e. soft-tissue, bone, gastro-intestinal, skin, female genital organs, other viscera and other sites), and to 2) histologic and 3) genetic groups based on guidelines developed by sarcoma specialists (see Additional File 1).

Age-standardized incidence rates (ASR) were estimated using direct standardization and were calculated using the population data for each age group and year supplied by the National Institute of Statistics and Economic Studies (www.insee.fr) and the European (ASR-E), Segi World (ASR-W), and the US (ASR-US) standard populations. The analyses presented here describe the overall ASR and the ASR by sex. Age-specific incidence rates are provided by age groups (0–14; 15–24; 25–39; 40–64; 65–74 and 75 and more) and by sex and presented in figures.

Time trends were calculated using Joinpoint Trend Analysis Software setting a maximum of a single Joinpoint (details in online supplementary material). The annual percent change (APC) with the 95% confidence interval (CI) was estimated according to topographic and histologic groups.

## Results

Over the 2010–13 period, sarcomas accounted for 1.3% (3942/307,862) of all malignant tumors diagnosed over the French registry area. The male/female ratio for overall sarcomas was 1.0 but ranged from 0.5 for angiosarcomas to 6.2 for Kaposi sarcomas (KS) (Table 1). The median age was 63 years (range: 0–106) with large intergroup variations. About 9% of subjects were under 24 years and 27% were older than 75 years. Almost half of the cases were soft tissue sarcomas (45%). The most frequent histological subtypes were undifferentiated or unclassified sarcomas (16%), leiomyosarcoma (14%) and GIST (13%). Sarcomas with complex genomics accounted for the most frequent molecular profile (40%).

**Table 1:** Gender distribution of sarcoma patients according to age and topographic, genomic and histologic groups. FRANCIM network data 2010–2013 (19 registries)

	***Male***		***Female***		***Overall***		***Sex ratio*** ***M/F***
	***n***	***%***	***n***	***%***	***n***	***%***	
**Age group (in years)**							
0–14	81	4.1	81	4.1	162	4.1	1.0
15–24	99	5.0	79	4.0	178	4.5	1.3
25–39	197	10.0	160	8.1	357	9.1	1.2
40–64	671	34.1	738	37.4	1409	35.7	0.9
65–74	376	19.1	379	19.2	755	19.2	1.0
75 and more	546	27.7	535	27.1	1081	27.4	1.0
**Sarcoma topographic groups**							
Soft tissue	972	49.3	812	41.2	1784	45.3	1.2
Bone	310	15.7	259	13.1	569	14.4	1.2
Skin	262	13.3	167	8.5	429	10.9	1.6
Viscera							
Gastro-intestinal organs	291	14.8	287	14.6	578	14.7	1.0
Female genital organs	–	–	282	14.3	282	7.2	–
Others visceral organs	102	5.2	129	6.5	231	5.9	0.8
Other anatomic sites	33	1.7	36	1.8	69	1.8	0.9
**Sarcoma genomic groups**							
Complex genomic alterations	723	36.6	847	43.0	1570	39.8	0.9
*MDM2* amplification	135	6.9	81	4.1	216	5.5	1.7
Mutations	274	13.9	276	14.0	550	14.0	1.0
Recurrent translocations	340	17.3	438	22.2	778	19.7	0.8
Undefined/Miscellaneous alterations	498	25.3	330	16.7	828	21.0	1.5
**Sarcoma histologic groups**							
Unclassified sarcoma^a^	327	16.6	308	15.6	635	16.1	1.1
Leiomyosarcoma	205	10.4	346	17.5	551	14.0	0.6
GIST	246	12.5	250	12.7	496	12.6	1.0
Liposarcoma	228	11.6	130	6.6	358	9.1	1.8
*Dedifferentiated liposarcoma*	*125*	*6.3*	*69*	*3.5*	*194*	*4.9*	*1.8*
*Round cell \ Myxoid liposarcoma*	*42*	*2.1*	*29*	*1.5*	*71*	*1.8*	*1.4*
*Pleomorphic liposarcoma*	*18*	*0.9*	*7*	*0.4*	*25*	*0.6*	*2.6*
*Liposarcoma NOS*	*43*	*2.2*	*25*	*1.3*	*68*	*1.7*	*1.7*
Chondrosarcoma	123	6.2	118	6.0	241	6.1	1.0
Dermatofibrosarcoma	101	5.1	124	6.3	225	5.7	0.8
Kaposi sarcoma	156	7.9	25	1.3	181	4.6	6.2
Angiosarcoma	54	2.7	115	5.8	169	4.3	0.5
Osteosarcoma	84	4.3	71	3.6	155	3.9	1.2
Ewing sarcoma	72	3.7	65	3.3	137	3.5	1.1
Myxofibrosarcoma	75	3.8	49	2.5	124	3.1	1.5
Rhabdomyosarcoma	66	3.4	51	2.6	117	3.0	1.3
*Embryonal rhabdomyosarcoma*	*27*	*1.4*	*16*	*0.8*	*43*	*1.1*	*1.7*
*Alveolar rhabdomyosarcoma*	*10*	*0.5*	*12*	*0.6*	*22*	*0.6*	*0.8*
*Pleomorphic rhabdomyosarcoma*	*14*	*0.7*	*7*	*0.4*	*21*	*0.5*	*2.0*
*Spindle cell rhabdomyosarcoma*	*7*	*0.4*	*7*	*0.4*	*14*	*0.4*	*1.0*
*Rhabdomyosarcoma NOS*	*8*	*0.4*	*9*	*0.5*	*17*	*0.4*	*0.9*
Nerve Sheath Tumors	38	1.9	44	2.2	82	2.1	0.9
Endometrial stromal sarcoma	–	–	81	4.1	81	2.1	–
Synovial sarcoma	37	1.9	40	2.0	77	2.0	0.9
*Spindle cell synovial sarcoma*	*19*	*1.0*	*20*	*1.0*	*39*	*1.0*	*1.0*
*Biphasic synovial sarcoma*	*3*	*0.1*	*7*	*0.3*	*10*	*0.2*	*0.4*
*Synovial sarcoma NOS*	*15*	*0.8*	*13*	*0.7*	*28*	*0.7*	*1.2*
Chordoma	40	2.0	27	1.4	67	1.7	1.5
Solitary fibrous tumor, malignant	33	1.7	33	1.7	66	1.7	1.0
Fibrosarcoma	15	0.8	16	0.8	31	0.8	0.9
Malignant myoepithelioma	12	0.6	11	0.6	23	0.6	1.1
Epithelioid haemangioendothelioma	9	0.5	11	0.6	20	0.5	0.8
Other (with fewer than 20 cases)	49	2.5	57	2.9	106	2.7	0.7
**Overall**	1970	100.0	1972	100.0	3942	100.0	1.0

^a^Unclassified sarcomas include: Sarcoma NOS (ICDO-88003), undifferentiated spindle cell sarcoma (ICDO-88013), undifferentiated pleomorphic sarcoma (ICDO-88023), undifferentiated round cell sarcoma (ICDO-88033), epithelioid sarcoma (ICDO-88043), undifferentiated sarcoma NOS (ICDO-88053)

The crude incidence rate and ASR-W of sarcomas were 7.4 and 5.0, respectively (Table 2). The ASR-W of soft tissue, bone and gastro-intestinal sarcomas were 2.1, 1.0 and 0.6, respectively. For the five most frequent histological subtypes, the ASR-W ranged from 0.3 to 0.7 with gender variations. For the two most frequent genomic profiles (over 60% of all sarcoma cases) the ASR-W was 1.9 for complex genomic and 1.3 for recurrent translocation events.

**Table 2:** Sarcoma crude and age-standardized incidence rate per 100,000 person-years according to topographic, genomic and histological groups by sex. FRANCIM network data 2010–2013 (19 registries)

	Median Age	Male				Female				Overall			
		CIR	ASR-W (segi)	ASR-E	ASR-US	CIR	ASR-W (segi)	ASR-E	ASR-US	CIR	ASR-W (segi)	ASR-E	ASR-US
**Sarcomas by topographic groups**													
Soft tissue	65	3.70	2.43	3.12	3.31	2.90	1.91	2.34	2.38	3.30	2.15	2.68	2.78
Bone	47	1.20	1.09	1.16	1.17	0.90	0.85	0.87	0.88	1.10	0.96	1.01	1.01
Skin	54	1.00	0.70	0.88	0.91	0.60	0.46	0.54	0.54	0.80	0.59	0.72	0.73
Viscera													
Gastro-intestinal organs	69	1.10	0.65	0.91	0.95	1.00	0.57	0.77	0.79	1.10	0.58	0.81	0.84
Female genital organs	62	–	–	–	–	1.00	0.62	0.82	0.81	1.00	0.62	0.82	0.81
Other visceral organs	65	0.40	0.26	0.33	0.33	0.50	0.28	0.36	0.37	0.40	0.26	0.34	0.35
Others anatomic sites	55	0.10	0.15	0.13	0.13	0.10	0.10	0.10	0.10	0.10	0.10	0.10	0.10
**Sarcomas by genomic groups**													
Complex genomic alterations	65	2.80	1.78	2.30	2.43	3.10	1.93	2.44	2.47	2.90	1.87	2.38	2.45
*MDM2* amplification	68	0.50	0.30	0.42	0.45	0.30	0.18	0.23	0.23	0.40	0.23	0.31	0.33
Mutations	68	1.10	0.66	0.87	0.90	1.00	0.58	0.76	0.77	1.00	0.62	0.80	0.83
Recurrent translocations	46	1.30	1.23	1.30	1.30	1.60	1.44	1.56	1.54	1.50	1.34	1.44	1.43
Undefined/Miscellaneous alterations	65	1.90	1.27	1.62	1.70	1.20	0.73	0.90	0.94	1.60	0.97	1.22	1.26
**Sarcomas by histologic groups**													
Unclassified sarcoma^a^	69	1.30	0.71	1.00	1.08	1.10	0.62	0.82	0.85	1.20	0.65	0.90	0.94
Leiomyosarcoma	66	0.80	0.43	0.62	0.68	1.30	0.74	0.99	0.99	1.00	0.58	0.79	0.81
GIST	69	0.90	0.52	0.75	0.78	0.90	0.47	0.65	0.67	0.90	0.50	0.70	0.72
Liposarcoma	67	0.90	0.48	0.68	0.72	0.50	0.28	0.37	0.37	0.70	0.38	0.51	0.53
*Dedifferentiated liposarcoma*	*69*	*0.50*	*0.24*	*0.35*	*0.38*	*0.30*	*0.11*	*0.16*	*0.16*	*0.40*	*0.17*	*0.25*	*0.26*
*Round cell \ Myxoid liposarcoma*	*51*	*0.20*	*0.13*	*0.16*	*0.16*	*0.10*	*0.09*	*0.11*	*0.11*	*0.10*	*0.10*	*0.12*	*0.12*
*Pleomorphic liposarcoma*	*73*	*0.10*	*0.03*	*0.04*	*0.05*	*0.00*	*0.00*	*0.00*	*0.00*	*0.00*	*0.01*	*0.02*	*0.02*
*Liposarcoma, NOS*	*67*	*0.20*	*0.09*	*0.13*	*0.14*	*0.10*	*0.04*	*0.05*	*0.06*	*0.10*	*0.05*	*0.08*	*0.08*
Chondrosarcoma	55	0.50	0.32	0.41	0.40	0.40	0.30	0.36	0.36	0.50	0.34	0.41	0.41
Dermatofibrosarcoma	44	0.40	0.31	0.35	0.35	0.50	0.40	0.45	0.44	0.40	0.37	0.42	0.41
Kaposi sarcoma	63	0.60	0.40	0.52	0.54	0.10	0.04	0.06	0.06	0.30	0.20	0.27	0.28
Angiosarcoma	73	0.20	0.10	0.15	0.16	0.40	0.17	0.25	0.27	0.30	0.16	0.23	0.25
Osteosarcoma	34	0.30	0.34	0.33	0.33	0.30	0.25	0.24	0.25	0.30	0.28	0.27	0.27
Ewing sarcoma	19	0.30	0.35	0.30	0.30	0.20	0.32	0.27	0.27	0.30	0.33	0.28	0.28
Myxofibrosarcoma	66	0.30	0.16	0.22	0.22	0.20	0.10	0.14	0.14	0.20	0.14	0.19	0.19
Rhabdomyosarcoma	25	0.30	0.26	0.24	0.25	0.20	0.21	0.18	0.18	0.20	0.25	0.22	0.23
*Embryonal rhabdomyosarcoma*	*12*	*0.10*	*0.15*	*0.11*	*0.11*	*0.10*	*0.09*	*0.06*	*0.06*	*0.10*	*0.11*	*0.08*	*0.08*
*Alveolar rhabdomyosarcoma*	*22*	*0.00*	*0.05*	*0.04*	*0.04*	*0.00*	*0.08*	*0.06*	*0.06*	*0.00*	*0.05*	*0.04*	*0.04*
*Pleomorphic rhabdomyosarcoma*	*69*	*0.10*	*0.02*	*0.03*	*0.03*	*0.00*	*0.01*	*0.01*	*0.01*	*0.00*	*0.01*	*0.02*	*0.02*
*Spindle cell rhabdomyosarcoma*	*40*	*0.00*	*0.00*	*0.00*	*0.01*	*0.00*	*0.03*	*0.02*	*0.02*	*0.00*	*0.00*	*0.00*	*0.00*
*Rhabdomyosarcoma NOS*	*64*	*0.00*	*0.01*	*0.01*	*0.01*	*0.00*	*0.01*	*0.02*	*0.02*	*0.00*	*0.01*	*0.02*	*0.02*
Nerve Sheath Tumors	55	0.10	0.10	0.13	0.13	0.20	0.12	0.15	0.15	0.20	0.12	0.15	0.15
Endometrial stromal sarcoma	62	–	–	–	–	0.30	0.17	0.23	0.23	0.30	0.17	0.23	0.23
Synovial sarcoma	47	0.10	0.16	0.17	0.17	0.10	0.13	0.15	0.14	0.10	0.15	0.16	0.16
*Spindle cell synovial sarcoma*	*49*	*0.1*	*0.07*	*0.05*	*0.05*	*0.10*	*0.05*	*0.06*	*0.06*	*0.10*	*0.05*	*0.07*	*0.07*
*Biphasic synovial sarcoma*	*44*	*0.0*	*0.00*	*0.00*	*0.00*	*0.00*	*0.02*	*0.01*	*0.01*	*0.00*	*0.00*	*0.00*	*0.00*
*Synovial sarcoma NOS*	*44*	*0.1*	*0.06*	*0.04*	*0.06*	*0.00*	*0.05*	*0.05*	*0.05*	*0.10*	*0.05*	*0.05*	*0.05*
Chordoma	61	0.20	0.11	0.14	0.15	0.10	0.06	0.08	0.08	0.10	0.09	0.12	0.12
Solitary fibrous tumor. Malignant	63	0.10	0.11	0.14	0.13	0.10	0.08	0.10	0.10	0.10	0.10	0.12	0.12
Fibrosarcoma	60	0.10	0.03	0.05	0.05	0.10	0.03	0.04	0.04	0.10	0.03	0.04	0.04
Malignant myoepithelioma	56	0.00	0.03	0.04	0.04	0.00	0.03	0.04	0.04	0.00	0.03	0.04	0.04
Epithelioid haemangioendothelioma	47	0.00	0.03	0.03	0.03	0.00	0.05	0.05	0.05	0.00	0.05	0.06	0.06
Other (with fewer than 20 cases)	46	0.20	0.19	0.17	0.18	0.20	0.19	0.20	0.20	0.20	0.20	0.20	0.20
**Total**	63	7.60	5.27	6.54	6.80	7.20	4.81	5.83	5.90	7.40	5.00	6.12	6.26

*Abreviations*: *GIST* Gastro-Intestinal Stromal Tumors, *CIR* Crude Incidence Rate per 100,000 persons-years, *ASR-W*, *ASR-E* and *ASR-US* Age-Standardized incidence Rate from three reference populations (W, World Segi; E, European; US, United-States)

^a^Unclassified sarcomas include: Sarcoma not otherwise specified (ICDO-88003), undifferentiated spindle cell sarcoma (ICDO-88013), undifferentiated pleomorphic sarcoma (ICDO-88023), undifferentiated round cell sarcoma (ICDO-88033), epithelioid sarcoma (ICDO-88043), undifferentiated sarcoma NOS (ICDO-88053)

**Fig. 1:**
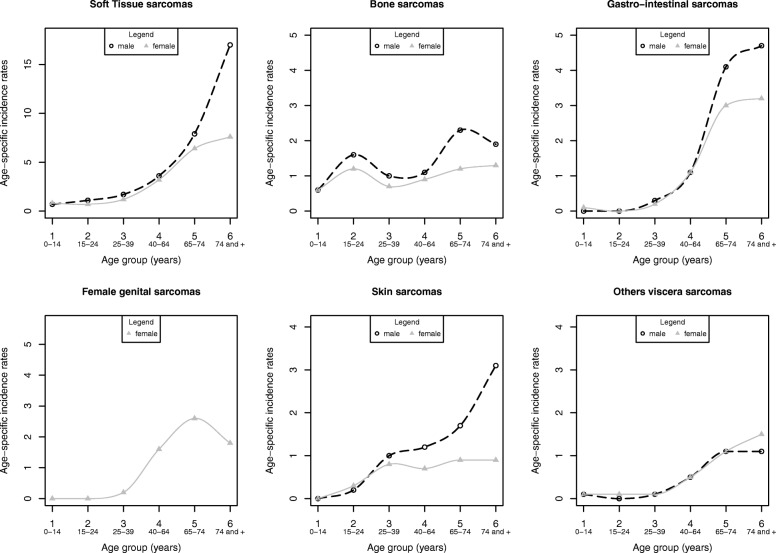
Age-specific incidence rates of sarcomas per 100,000 person-years according to topographic groups. FRANCIM network data 2010–2013 (19 registries)

The overall sarcoma incidence peaked at 22 in patients aged 75 or over (data not shown). Age-specific rates for soft tissue, viscera and skin sarcomas were relatively stable among patients aged between 0 and 40 years, and then increased with age (Fig. 1). This increase was less pronounced in women. In men, bone sarcomas presented a biphasic profile with a first peak in young people between 15 and 25 years of age and a second peak in adults aged between 65 and 74 years of age. With respect to histological subtypes, age-specific incidence rates had various profiles (see Additional File 2). According to the genomic profile, the incidence increased steadily with age, except for tumors harboring recurrent translocations and *MDM2* amplification among women (see Additional File 3).

The ASR-W for all sarcomas increased between 2000 and 2005 (APC = 3.6%), and remained stable since 2005 (non-significant APC, Table 3). According to the anatomic site, the ASR-W decreased for skin sarcomas (APC = -2.0%) and female genital tumors between 2005 and 2013 (APC = -2.2%). Stratifying by major histological subtypes, the ASR-W increased for GIST (APC = 3.7%), chondrosarcoma (APC = 4.1%), myxofibrosarcoma (8.2%) and solitary fibrous tumors (12.2%) and decreased for leiomyosarcoma (APC = -2.6%), Kaposi sarcoma (− 4.1%) and fibrosarcoma (APC = -9.2%). All trend figures are provided in the online supplementary material (see Additional Files 4 and 5).

**Table 3:** Annual percentage change of world age-standardized incidence rate by topographic groups, histologic types. FRANCIM network data 2000–2013 (11 registries)

	n	Joinpoint	APC	95% CI
**Sarcomas by topographic groups**				
Soft Tissue	3766		0.8	(−0.4; 2.0)
Bone	1193		1.2	(−0.4; 2.9)
Skin	1062		**-2.0**^*a*^	(−3.5; −0.4)
Viscera tumors organs				
Gastro-intestinal organs	1053		1.5	(−0.3; 3.3)
Female genital organs	297	2000–2005	4.2	(−4.2; 13.2)
	376	2005–2013	**−6.7**^*a*^	(−10.4; −2.7)
Other visceral organs	540		−1.7	(−5.1; 1.8)
Other anatomic sites	171		1.0	(−3.4; 5.6)
**Sarcomas by histologic groups**				
Unclassified sarcoma	1513		−1.6	(−3.6; 0.3)
Leiomyosarcoma	1281		**−2.6**^*a*^	(−4.6; −0.6)
GIST	822		**3.7**^*a*^	(0.8; 6.8)
Liposarcoma	713		1.3	(−1.1; 3.7)
Dermatofibrosarcoma	496		0.6	(−1.4; 2.7)
Chondrosarcoma	454		**4.1**^*a*^	(1.6; 6.6)
Kaposi sarcoma	419		**−4.1**^*a*^	(−6.8; −1.4)
Osteosarcoma	359		−0.6	(−3.7; 2.6)
Angiosarcoma	335		2.2	(−1.2; 5.7)
Ewing sarcoma	330		−0.2	(−4.1; 3.8)
Rhabdomyosarcoma	286		−1.1	(−6.2; 4.4)
Others (with fewer than 20 cases)	226		4.1	(−1.3; 9.8)
Synovial sarcoma	219		1.2	(−4.2; 6.9)
Nerve Sheath Tumors	191		−0.1	(−4.3; 4.4)
Myxofibrosarcoma	183		**8.2**^*a*^	(0.4; 16.6)
Endometrial stromal sarcoma	173		−3.7	(−7.4; 0.1)
Fibrosarcoma	151		**−9.2**^*a*^	(−15.7; −2.3)
Chordoma	126		0.8	(−4.8; 6.6)
Solitary fibrous tumor. Malignant	102		**12.2**^*a*^	(6.2; 18.5)
Epithelioid haemangioendothelioma	55		–	–
Myoepithelial carcinoma	24		–	–
**Total**	3359	2000–2005	**3.6**^*a*^	(0.2; 7.1)
	5099	2005–2013	−1.4	(−2.9; 0.1)

Note. Joinpoint = years when statistically significant changes in incidence trend occurred

*APC* Annual Percent Change, *CI* Confidence Interval

^a^Indicates that the APC is significantly different from 0 at the alpha = 0.05 level

## Discussion

In this study, we precisely described the incidence of sarcomas according to different classifications (anatomic, histologic and genetic) using data from population-based cancer registries. To our knowledge, this is one of the first reports on sarcomas based on a systematic pathological review of these cancers while taking into account the updated sarcoma classifications.

In this study, sarcomas accounted for 1.3% of all malignant tumors (1.1% for soft tissue -including skin and viscera- and 0.2% for bone) and had an ASR-E of 6.1 per 100,000 person-years over the 2010–2013 period (European population standard). The ASR-E was slightly higher than that reported in Europe [12]. Data comparison between countries is difficult due to the heterogeneity of sarcoma definition used as inclusion criteria. This heterogeneity is mainly related to some analysis characteristics: i) certain specific histological subtypes are not consistently included in analyses (e.g. Kaposi sarcoma or dermatofibroma sarcoma); ii) some studies consider adults and children separately, while others mix them; and iii) anatomic sites may be limited to specific sites such as STS. The current approach to describe sarcomas using registry data based on expert recommendations are expected to better follow epidemiological indicators and to carry out reliable comparisons between countries.

With respect to the anatomic site, ASR-E for STS (2.7) in our study was below most published international incidence rates. This may be explained by the exclusion of visceral sarcomas of soft tissue and the different description of well-differentiated liposarcoma compared to the WHO 2013 classification. In the current study, ASR-Ws for bone sarcomas among males and females (1.1 and 0.9 respectively) were close to those recently reported in five continents (2010–13 period, ASR-W 0.8–1.2 in males and 0.5–1.0 in females) [13]. For visceral sarcomas, the comparison between studies with inclusion periods close to that in the present study showed ASR-E similar to ours [8, 14]. In contrast, the ASR was greater than that reported in the RARECARE project, which may be due to differences in the definition of visceral sarcomas (GIST not included) [14].

The comparison of ASRs for main histologic groups between studies with a shorter inclusion period showed that the ASR-E for leiomyosarcoma (0.8; 0.6 for males and 1.0 for females) was greater than that reported in France (0.6) and was similar to that reported in three European regions (0.5 for males and 1.0 for females) [8, 14]. ASR-E for liposarcoma in our study (0.5; 0.7 for males and 0.4 for females), was lower than that reported in France (0.8) and in three European regions (1.06 for males and 0.59 for females), which may be attributed to differences in the definition of liposarcoma as inclusion criteria [8, 14]. In our study, we found an ASR-W for osteosarcoma slightly lower than that of chondrosarcoma (0.28 versus 0.34). For male, ASR-W was equivalent (0.34 versus 0.32). A recent population-based study from Swiss cancer registries showed similar results [15]. In contrast, others studies based on older inclusion period of sarcoma diagnosis found an ASR-W slightly higher for osteosarcoma [8, 16]. However, looking at the trend in our study (Additional File 5), we can notice that the ASR-W of osteosarcoma was actually higher over the period 2000–2005 than the ASR-W of chondrosarcoma in accordance with these studies. The increasing trend in the ASR of chondrosarcoma and the stabilization of the ASR of osteosarcomas may logically explain why the incidence of chondrosarcomas has been higher than that of osteosarcomas in recent years.

Molecular biology of sarcomas, available for diagnosis in France since 2010 is a complementary approach and has led to a molecular classification for sarcomas [17]. For the first time, we provided ASR at national level and showed molecular profiles by age groups.

This study provides the first time trend analysis of sarcomas in France and shows that ASR-W for sarcomas increased between 2000 and 2005 (APC = 3.6%) and stabilized from 2005. The current study has not shown an increase in ASR-W for soft-tissue sarcomas. This is in contrast to reports in others countries covering different periods: in the United States APC was 1.2% for males and 0.8% for females between 1978 and 2001, in Japan APC was 0.6% between 1978 and 2007 and in Serbia APC was 0.77% between 1985 and 2009 [18–20]. We report a significant decrease in incidence for skin sarcomas over the study period and for female genital sarcomas since 2005. Some histological subtypes have shown a significant decrease over the study period: leiomyosarcoma, KS and fibrosarcoma. The decline for KS has also been described in the population from the United States over the same period [21]. These changes are consistent with the improvement in access for antiretroviral therapy among HIV-infected patients and the declining AIDS incidence in developed countries. The decrease in incidence of leiomyosarcoma and fibrosarcoma could be explained by a histological classification published by the WHO in 2002 that includes new data of immunohistochemistry and new histological subtypes. Similarly, we report an increase in incidence of GIST, likely related to the introduction in the early 2000s of an immunohistochemical diagnostic test specific to GIST tumors (*KIT*-activating mutations). Further, the increase in GIST was more noticeable before 2005 and stabilised after 2005. The time trend analysis also revealed a significant increase for chondrogenic sarcomas (APC = 4.4%). Such increase has been reported in a study from the United States including only women (1976–2005) [20], whereas a study from the United Kingdom showed the same trend in incidence for both sexes (1988–2007) [13]. The strongest hypothesis to explain the increased risk of chondrogenic sarcoma in women is the introduction of exogenous estrogen exposures (oral contraceptives, hormone therapy), whereas other factors has to be identified in men [13, 16].

The different incidence trends for sarcomas reported over the world may partly be explained by variations in diagnosis practices and the classification used. The impact of environmental factors in the etiology of these cancers may also be a point at issue. However, the large heterogeneity of histological subtypes and the rarity of sarcomas prevent examining this association and drawing conclusions from existing environmental epidemiological studies. A national French study on the etiology of sarcomas (Etiosarc) has been launched to study the possible effect of environmental factors [22].

A major strength in this study is that the incidence of sarcomas was estimated using the 2013 WHO classification [1]. Whenever possible, registry data was converted to the latest classification to take into account changes and evolutions between different classifications (e.g. new morphological terms, obsolete morphological codes and terms).

Moreover, this study is the first to describe sarcomas in a geographic area where an expert sarcoma pathologist reviews the pathologic diagnosis. Contrary to imperfectly estimated sarcoma incidence rates, this review allows to provide a consistent incidence of sarcomas. A French study, confirmed these results and indicated that 45% of sarcomas are misclassified at initial diagnosis and that 19% have complete discordance [3]. For this reason, the review for sarcoma diagnosis is necessary to estimate a consistent incidence and more so for the different subgroups. In France, the second review was based on voluntary participation before the year 2010. Thereby, we cannot be certain that the review was obtained for all sarcomas in the period 2000–2010, even if significant efforts were made by French sarcoma network in order that pathologists systematically send slides of any newly diagnosed of sarcomas. For this reason, the estimated incidence over the 2010–2013 seems to be most relevant and reliable.

## Conclusion

This study provided the opportunity to precisely describe the incidence of sarcomas according to three different groups (anatomic, histologic and genetic) defined by sarcoma specialists using data from population-based cancer registries. To our knowledge, this study is the first to report sarcoma incidence based on a systematic pathological review of these cancers and taking into account the updated sarcoma classifications. Due to literature paucity on sarcomas, future studies using data from population-based cancer registries will have to consider a strict inclusion criterion presented in our study to better describe and compare data between countries. The molecular classification will be useful for etiological studies as incidence studies.

## Supplementary information

**Additional file 1.** Complementary information on data collection and statistical analyses.

**Additional file 2: Figure S1.** Age-specific incidence rates of sarcomas per 100,000 person-years according to histologic groups. FRANCIM network data 2010–2013 (19 registries).

**Additional file 3: Figure S*2*.** Age-specific incidence rates of sarcomas per 100,000 person-years according to genomic groups. FRANCIM network data 2010–2013 (19 registries).

**Additional file 4: Figure S3.** Sarcoma trends and annual percentage change (APC) of world age-standardized incidence rate according to topographic group. FRANCIM network data 2000–2013 (11 registries).

**Additional file 5: Figure S4.** Sarcoma trends and annual percentage change (APC) of world age-standardized incidence rate according to histologic group. FRANCIM network data 2000–2013 (11 registries).

## Data Availability

The datasets analyzed during the current study are not publicly available due to national regulations. Permission to use French cancer registry data was provided by the National Cancer Institute after consultation with the data protection authority.

## Abbreviations

APC: Annual percentage change
ASR: Age-standardized incidence rates
CI: Confident interval
GIST: Gastro-intestinal stromal tumors
ICD-O-3: International Classification of Diseases for Oncology, third edition
KS: Kaposi sarcoma
