# Supplementary material for: Incidence and time trends of sarcoma (2000–2013): results from the French network of cancer registries (FRANCIM)
Source: BMC Cancer. 2020 Mar 6;20:190. doi: 10.1186/s12885-020-6683-0 (PMC7059296; doi:10.1186/s12885-020-6683-0)
Supplement: Supplementary file 1 — Additional file 1. Complementary information on data collection and statistical analyses. [file 12885_2020_6683_MOESM1_ESM.docx]

**Online supplementary materials**

**Complementary information on data collection and statistical analyses (p2)**

**Guidelines for the analysis of sarcomas from registry data (p4)**

**Complementary information on data collection and statistical analyses**

**Cancer Registry Network**

The FRANCIM network coordinates all population-based cancer registries in France and harmonizes cancer collection and data quality using the “*IARCcrgTools*” software developed by the International Agency for Research on Cancer. Quality assessment is also performed at the registry level using local control. The FRANCIM database comprises data from 19 general cancer registries (Bas-Rhin, Calvados, Charente, Charente Maritime, Deux-Sèvres, Doubs, Gironde, Haut-Rhin, Haute-Vienne, Hérault, Isère, Lille, Loire Atlantique, Manche, Somme, Tarn, Territoire de Belfort, Vendée and Haute-Vienne) covering around 20% of the whole French metropolitan population. The quality and completeness of all French registries are examined every four years by an independent and national expert committee named by national authorities.

**Quality control of the diagnosis of sarcomas**

Owing to a high risk of initial diagnosis disagreement, a second review by expert pathologist is essential. The network involved 22 French pathology centers, including three coordinating national centers and 19 regional centers across the country. These networks have been previously described [1].

Certain alignments could not be performed: ten morphological terms not described in this updated classification (e.g. sarcoma NOS, periosteal fibrosarcoma, fascial fibrosarcoma) have been maintained for analyses. Conversely, well-differentiated liposarcoma and chondroblastoma have been changed from malignant to borderline diseases. In the same way, behaviors for dermatofibrosarcoma protuberans and pigmented dermatofibrosarcoma protuberans have been also changed from malignant to borderline with henceforth, only fibrosarcomatous dermatofibrosarcoma protuberans which is coded as malignant behavior. In our analyses, we chose to keep all dermatofibrosarcomas. Indeed, we cannot differentiate whether it is a borderline or malignant case. Besides, endometrial stromal sarcoma NOS (89303), low grade endometrial stromal sarcoma (89313) and stromal sarcoma (89353), not described in the WHO 2013 classification, have been also included.

**Statistical analyses**

Time trends were calculated using the Joinpoint Trend Analysis Software (version 4.6.0.0) (https://surveillance.cancer.gov/joinpoint/). Time trends in the incidence were estimated by the annual percent change (APC), calculated as the slope of the linear regression used to model the natural logarithm of the ASR (Segi World) as a function of the calendar year [2]. Given the relatively short study period (14 years), the software was set to define a maximum of a single Joinpoint. The software estimates the APC of each segment between Joinpoints and test whether it is significantly different from zero using a MonteCarlo permutation method [3]. The 14-year incidence time trends were evaluated using the estimated average APC with a 95% confidence interval (CI) according to topographic or histologic groups.

**References**

1. Honoré C, Méeus P, Stoeckle E, Bonvalot S. Soft tissue sarcoma in France in 2015: Epidemiology, classification and organization of clinical care. J. Visc. Surg. 2015; 152(4):223–230.

2. Noone A, Howlader N, Krapcho M et al. SEER Cancer Statistics Review, 1975-2015, National Cancer Institute. Bethesda, MD, https://seer.cancer.gov/csr/1975_2015/, based on November 2017 SEER data submission, posted to the SEER web site, April 2018. .

3. Kim HJ, Fay MP, Feuer EJ, Midthune DN. Permutation tests for joinpoint regression with applications to cancer rates. Stat. Med. 2000; 19(3):335–351.

**Guidelines for the analysis of sarcomas from registry data**

Most cancer registries record sarcoma cases according to the ICD-O-3 and do not take into account the recent 2013 World Health Organization (WHO) Classification of Tumors of Soft Tissue and Bone.

A working group of medical experts (pathologists, clinicians and epidemiologists) was consulted with the aim of proposing recommendations for the analysis of sarcomas from registry data.

The experts' opinion was solicited to validate:

- A list of topographical groups to describe sarcomas from registry data.
- A list of morphology codes to describe sarcomas based on the ICD-O-3 classification and the 2013 WHO Classification of Tumors of Soft Tissue and Bone.
- A list of genomic alterations from sarcoma subtypes.

This section of the supplementary material provides the three lists proposed by the group of experts for the epidemiological description of sarcomas for clinical purposes.

Table A lists the topographical groups used to describe sarcomas.

Tables B to I present the list of morphology codes and their evolution over time based on the latest 2013 World Health Organization (WHO) Classification of Tumors of Soft Tissue and Bone standard.

Table J presents the morphology codes according to genomic alterations.

Table A. Topographical groups used to describe sarcomas.

| **Topographical groups** | **Topographical codes (ICD-O-3)** |
| --- | --- |
| **1 – Soft tissue sarcomas** | - Lip (C00)  - Tongue (C01-02)  - Gum (C03)  - Floor of mouth (C04)  - Other and unspecified parts of mouth (C05-06)  - Oropharynx (C09-10)  - Nasopharynx (C11)  - Hypopharynx (C12-13)  - Other and ill-defined sites in lip, oral cavity and pharynx (C14)  - Nasal cavity and middle ear and accessory sinuses (C30-31)  - Larynx (C32)  - Mediastinum (C38.1-38.3)  - Peripheral nerves and autonomic nervous system (C47)  - Retroperitoneum and peritoneum (C48)  - Connective, subcutaneous and other soft tissues (C49)  - Overlapping lesion of eye and adnexa (C69.8) |
| **2 – Bone sarcomas** | - Bone (C40-41) |
| **3 – Viscera sarcomas** |  |
| 3a – Gastro-intestinal sarcomas | **-** Esophagus (C15)  - Stomach (C16)  - Small intestine(C17)  - Colon and rectum (C18-20)  - Anus and anal canal (C21)  - Liver and intrahepatic bile ducts (C22)  - Gallbladder and extrahepatic biliary tract (C23-24)  - Pancreas (C25)  - Other and ill-defined digestive organs (C26) |
| 3b – Female genital sarcomas | **-** Vulva and vagina (C51-52)  - Cervix and corpus uteri (C53-54)  - Corpus NOS (C55)  - Ovary (C56)  - Other and unspecified female genital organs (C57)  - Placenta (C58) |
| 3c – Other viscera sarcomas | **-** Salivary glands (C07-C08)  - Trachea, bronchus and lung (C33-34)  - Thymus (C37)  - Heart (C38.0)  - Pleura SAI (C38.4)  - Breast (C50)  - Penis, other and unspecified male genital organs (C60, C63)  - Prostate gland (C61)  - Testis (C62)  - Urinary system (C64-C68)  - Eyes except overlapping lesion of eye and adnexa (C69 except C69.8)  - Thyroïd gland (C73)  - Adrenal gland and other endocrine glands (C74-75) |
| **4 – Skin sarcomas** | - Skin (C44) |
| **6 – Other sites** | - Overlapping lesion of heart, mediastinum and pleura (C38.8)  - Spleen (C42.2)  - Meninges (C70)  - Brain (C71)  - Spinal cord, cranial nerves, and other parts of central nervous system (C72)  - Other and ill-defined sites(C76)  - Lymph nodes (C77) |

**Table B**. List of morphology terms / codes unchanged between the ICD-O-3 classification and the 2013 World Health Organization (WHO) Classification of Tumors of Soft Tissue and Bone.

| **Code ICD-O-3** | **WHO Classification** | **Morphology terms (WHO 2013)** |
| --- | --- | --- |
| 87113 | Soft-tissue | Malignant glomus tumor |
| 88053 | Soft-tissue | Undifferentiated sarcoma NOS |
| 88063 | Soft-tissue | Desmoplastic small round cell tumor |
| 88113 | Soft-tissue | Myxofibrosarcoma |
| 88143 | Soft-tissue | Infantile fibrosarcoma |
| 88153 | Soft-tissue | Solitary fibrous tumor, malignant |
| 88403 | Soft-tissue | Low-grade fibromyxoid sarcoma / Sclerosing epithelioid fibrosarcoma |
| 88523 | Soft-tissue | Myxoid liposarcoma |
| 88543 | Soft-tissue | Pleomorphic liposarcoma |
| 88583 | Soft-tissue | Dedifferentiated liposarcoma |
| 89013 | Soft-tissue | Pleomorphic rhabdomyosarcoma |
| 89103 | Soft-tissue | Embryonal rhabdomyosarcoma (incl. botryoid, anaplastic) |
| 89123 | Soft-tissue | Spindle cell / sclerosing rhabdomyosarcoma |
| 89203 | Soft-tissue | Alveolar rhabdomyosarcoma (incl. solid, anaplastic) |
| 89213 | Soft-tissue | Ectomesenchymoma |
| 89363 | Soft-tissue | Gastrointestinal stromal tumor, malignant |
| 89403 | Soft-tissue | Mixed tumor NOS, malignant |
| 89823 | Soft-tissue | Myoepithelial carcinoma |
| 90403 | Soft-tissue | Synovial sarcoma NOS |
| 90413 | Soft-tissue | Synovial sarcoma, spindle cell |
| 90433 | Soft-tissue | Synovial sarcoma, biphasic |
| 90443 | Soft-tissue | Clear cell sarcoma of soft tissue |
| 91403 | Soft-tissue | Kaposi sarcoma |
| 91813 | Bone | Chondroblastic osteosarcoma |
| 91823 | Bone | Fibroblastic osteosarcoma |
| 91833 | Bone | Telangiectatic osteosarcoma |
| 91853 | Bone | Small cell osteosarcoma |
| 91923 | Bone | Parosteal osteosarcoma |
| 91933 | Bone | Periosteal osteosarcoma |
| 91943 | Bone | High-grade surface osteosarcoma |
| 92423 | Bone | Clear cell chondrosarcoma |
| 92433 | Bone | Dedifferentiated chondrosarcoma |
| 92503 | Bone | Malignancy in giant cell tumor of bone |
| 92523 | Soft-tissue | Tenosynovial giant cell tumor malignant |
| 92613 | Bone | Adamantinoma |
| 93703 | Bone | Chordoma, NOS |
| 93713 | Bone | Chondroïd chordoma |
| 93723 | Bone | Dedifferentiated chordoma |
| 95403 | Soft-tissue | Malignant peripheral nerve sheath tumor |
| 95713 | Soft-tissue | Malignant perineurioma |
| 95613 | Soft-tissue | Malignant Triton tumor |
| 95803 | Soft-tissue | Malignant granular cell tumor |
| 95813 | Soft-tissue | Alveolar soft-part sarcoma |

**Table C**. List of new morphology terms included in the updated WHO 2013 classification.

| **Code ICD-O-3** | **WHO Classification** | **Morphology terms (ICD-O-3)** | **Morphology terms (WHO 2013)** |
| --- | --- | --- | --- |
| 88013 | Soft-tissue | Spindle cell sarcoma | Undifferentiated spindle cell sarcoma |
| 88023 | Soft-tissue | [Pleomorphic cell sarcoma](http://codes.iarc.fr/code/3299) / Giant cell sarcoma | Undifferentiated pleomorphic sarcoma |
| 88033 | Soft-tissue | Small/round cell sarcoma | Undifferentiated round cell sarcoma |
| 88043 | Soft-tissue | Epithelioid sarcoma | Undifferentiated epithelioid sarcoma |
| 88103 | Soft-tissue | [Fibrosarcoma, NOS](http://codes.iarc.fr/code/3309) | Adult fibrosarcoma |
| 88103 | Bone | [Fibrosarcoma, NOS](http://codes.iarc.fr/code/3309) | Fibrosarcoma of bone |
| 88323* | Soft-tissue | Dermatofibrosarcoma, NOS (C44.-) | Fibrosarcomatous dermatofibrosarcoma protuberans |
| 88503 | Soft-tissue | Liposarcoma, NOS | Liposarcoma, not otherwise specified |
| 88503 | Bone | Liposarcoma, NOS | Liposarcoma of bone |
| 89633 | Soft-tissue | [Malignant rhabdoid tumor](http://codes.iarc.fr/code/3497) | Extra-renal rhabdoid tumor |
| 88903 | Soft-tissue | Leiomyosarcoma, NOS | Leiomyosarcoma (excluding skin) |
| 88903 | Bone | Leiomyosarcoma, NOS | Leiomyosarcoma of bone |
| 89903 | Soft-tissue | [Mesenchymoma, malignant](http://codes.iarc.fr/code/3523) | Phosphaturic mesenchymal tumor, malignant |
| 91803 | Soft-tissue | [Osteosarcoma, NOS (C40._,C41._)](http://codes.iarc.fr/code/3723) | Conventional osteosarcoma |
| 91803 | Bone | [Osteosarcoma, NOS (C40._,C41._)](http://codes.iarc.fr/code/3723) | Extraskeletal osteosarcoma |
| 91873 | Bone | Intraosseous well differentiated osteosarcoma (C40. _,C41._) | Low-grade central osteosarcoma |
| 91203 | Soft-tissue | [Haemangiosarcoma](http://codes.iarc.fr/code/3671)/[Angiosarcoma](http://codes.iarc.fr/code/3671) | Angiosarcoma of soft tissue |
| 91203 | Bone | [Haemangiosarcoma](http://codes.iarc.fr/code/3671)/[Angiosarcoma](http://codes.iarc.fr/code/3671) | Angiosarcoma |
| 91843 | Bone | Osteosarcoma in Paget disease of bone (C40.-, C41.-) | Secondary osteosarcoma |
| 92203 | Bone | Chondrosarcoma, NOS (C40.-, C41.-) | Chondrosarcoma grade II, grade III |
| 92313 | Soft-tissue | Myxoid Chondrosarcoma | Extraskeletal myxoid chondrosarcoma |
| 92403 | Bone | Mesenchymal chondrosarcoma | Mesenchymal chondrosarcoma |
| 92403 | Soft-tissue | Mesenchymal chondrosarcoma | Extraskeletal mesenchymal chondrosarcoma |
| 93643 | Soft-tissue | [Peripheral neuroectodermal tumor](http://codes.iarc.fr/code/3849) | Extraskeletal Ewing sarcoma |
| 93643 | Bone | [Peripheral neuroectodermal tumor](http://codes.iarc.fr/code/3849) | Ewing sarcoma |

*Dermatofibrosarcoma (NOS and dermatofibrosarcoma protuberans) behavior has been changed from "malignant" to "borderline malignancy". Only fibrosarcomatous dermatofibrosarcoma protuberans must be code with malignant behavior.

**Table D**. List of obsolete ICD-O-3 morphological codes and their alignment with the 2013 World Health Organization (WHO) Classification of Tumors of Soft Tissue and Bone.

| **WHO Classification** | **Code ICD-O-3** | **Morphology terms (ICD-O-3)** | **Alignement new code (OMS 2013)** | **Morphology terms (WHO 2013)** |
| --- | --- | --- | --- | --- |
| Soft-tissue | 87103 | [Glomangiosarcoma](http://codes.iarc.fr/code/3225) | 87113 | Malignant glomus tumor |
| Soft-tissue | 88303 | [Malignant fibrous histiocytoma](http://codes.iarc.fr/code/3346) | 88023 | Undifferentiated pleomorphic sarcoma |
| Bone | 88303 | [Malignant fibrous histiocytoma](http://codes.iarc.fr/code/3346) | 88303 | Undifferentiated high-grade pleomorphic sarcoma of bone |
| Soft-tissue | 88533 | [Round cell liposarcoma](http://codes.iarc.fr/code/3385) | 88523 | Round cell \ Myxoid liposarcoma |
| Soft-tissue | 88553 | Mixed liposarcoma | 88523 | Round cell \ Myxoid liposarcoma |
| Soft-tissue | 88573 | F[ibroblastic liposarcoma](http://codes.iarc.fr/code/3393) | 88513 / 88583 | Well differentiated / dedifferentiated liposarcoma |
| Soft-tissue | 88913 | [Epithelioid leiomyosarcoma](http://codes.iarc.fr/code/3417) | 88903 | Leiomyosarcoma (excluding skin) |
| Digestive | 88913 | [Epithelioid leiomyosarcoma](http://codes.iarc.fr/code/3417) | 88363 | Gastrointestinal stromal tumor, malignant |
| Soft-tissue | 88943 | [Angiomyosarcoma](http://codes.iarc.fr/code/3426) | 91203 | Angiosarcoma of soft tissue |
| Bone | 88943 | [Angiomyosarcoma](http://codes.iarc.fr/code/3426) | 91203 | Angiosarcoma |
| Soft-tissue | 88953 | [Myosarcoma](http://codes.iarc.fr/code/3428) | 88903 | Leiomyosarcoma (excluding skin) |
| Soft-tissue | 88963 | [Myxoid leiomyosarcoma](http://codes.iarc.fr/code/3429) | 88903 | Leiomyosarcoma (excluding skin) |
| Soft-tissue | 89023 | [Mixed type rhabdomyosarcoma](http://codes.iarc.fr/code/3438) | 89103/89203 | [Embryonal rhabdomyosarcoma, NOS](http://codes.iarc.fr/code/3444) or Alveolar rhabdomyosarcoma (incl. solid, anaplastic) |
| Soft-tissue | 91303 | [Hemangioendothelioma, malignant](http://codes.iarc.fr/code/3684) | 91333 | Epithelioid haemangioendothelioma |
| Bone | 91303 | [Hemangioendothelioma, malignant](http://codes.iarc.fr/code/3684) | 91333 | Epithelioid haemangioendothelioma |
| Soft-tissue | 91503 | [Hemangiopericytoma, malignant](http://codes.iarc.fr/code/3706) | 88153 | Solitary fibrous tumor, malignant |
| Soft-tissue | 91703 | L[ymphangiosarcoma](http://codes.iarc.fr/code/3710) | 91203 | Angiosarcoma of soft tissue |
| Bone | 91863 | Central osteosarcoma | 91873 | Low-grade central osteosarcoma |
| Bone | 92213 | Ju[xtacortical chondroma (C40._,C41._)](http://codes.iarc.fr/code/3759) | 92203 | Chondrosarcoma grade II, grade III |
| Bone | 92603 | [Ewing sarcoma](http://codes.iarc.fr/code/3784) | 93643 | Ewing sarcoma |
| Soft-tissue | 92603 | [Ewing sarcoma](http://codes.iarc.fr/code/3784) | 93643 | Extraskeletal Ewing sarcoma |
| Bone | 93653 | Askin tumor | 93643 | Ewing sarcoma |
| Soft-tissue | 93653 | Askin tumor | 93643 | Extraskeletal Ewing sarcoma |
| Soft-tissue | 94733 | [Primitive neuroectodermal tumor, NOS](http://codes.iarc.fr/code/4370) | 93643 | Extraskeletal Ewing sarcoma |
| Soft-tissue | 95603 | Neurilemoma, malignant | 95403 | Malignant peripheral nerve sheath tumor |

**Table E**. List of new codes and new terms to describe malignant behavior not present in the ICD-O-3 classification.

| **WHO Classification** | **Morphology codes (WHO 2013)** | **Morphology terms (WHO 2013)** |
| --- | --- | --- |
| Soft-tissue | 87143 | PEComa NOS, malignant |
| Soft-tissue | 91373 | Intimal sarcoma |

**Table F**. List of terms to describe malignant behavior not present in the ICD-O-3 classification.

| **WHO Classification** | **Morphology codes (WHO 2013)** | **Morphology terms (WHO 2013)** |
| --- | --- | --- |
| Soft-tissue | 95423 | Epithelioid malignant nerve sheath tumor |
| Soft-tissue | 88253 | Low-grade myofibroblastic sarcoma |
| Soft-tissue | 88423 | Ossifying fibromyxoid tumor, malignant |

**Table G**. List of terms of morphology whose behavior has been changed from "malignant" to "borderline malignancy".

| **Morphology codes (WHO 2013)** | **Morphology codes (ICD-O-3)** | **Morphology terms (ICD-O-3)** | **Morphology codes (WHO 2013)** | **Morphology terms (WHO 2013)** |
| --- | --- | --- | --- | --- |
| Soft-tissue | 88513 | [Liposarcoma, well differentiated](http://codes.iarc.fr/code/3376) | 88501 | Atypical lipomatous tumor |
| Soft-tissue | 88323* | [Dermatofibrosarcoma protuberans (C44. _)](http://codes.iarc.fr/code/3359) | 88321 | Dermatofibrosarcoma protuberans |
| Soft-tissue | 88333* | [Pigmented dermatofibrosarcoma protuberans (C44._)](http://codes.iarc.fr/code/3359) | 88331 | Pigmented dermatofibrosarcoma protuberans |
| Bone | 92303 | Chondroblastoma malignant (C40._, C41._) | 92301 | Chondroblastoma |

*Dermatofibrosarcoma (NOS and dermatofibrosarcoma protuberans) behavior has been changed from "malignant" to "borderline malignancy". Only fibrosarcomatous dermatofibrosarcoma protuberans must be code with malignant behavior.

**Table H**. List of terms/codes not described in the WHO 2013 classification update but retained for the sarcoma description.

| **Code ICD-O-3** | **Morphology terms (ICD-O-3)** |
| --- | --- |
| 88003 | Sarcoma, NOS |
| 88123 | [Periosteal fibrosarcoma (C40._, C41._)](http://codes.iarc.fr/code/3315) |
| 88133 | [Fascial fibrosarcoma](http://codes.iarc.fr/code/3318) |
| 89003 | Rhabdomyosarcoma, NOS |
| 89643 | Clear cell sarcoma of kidney (C64.9) |
| 89913 | Embryonal sarcoma |
| 90423 | Synovial sarcoma, epithelioid cell |
| 91953 | In[tracortical osteosarcoma (C40._,C41._)](http://codes.iarc.fr/code/3744) |
| 92513 | [Giant cell tumor of soft parts, NOS](http://codes.iarc.fr/code/3777) |
| 95083 | Aty[pical teratoid/rhabdoid tumor (C71._)](http://codes.iarc.fr/code/3941) |

**Table I**. List of morphology terms / codes associated with other sites not described in the WHO 2013 classification.

| **Code ICD-O-3** | **Morphology terms (ICD-O-3)** |
| --- | --- |
| 89303 | [Endometrial stromal sarcoma, NOS (C54.1)](http://codes.iarc.fr/code/3453) |
| 89313 | End[ometrial stromal sarcoma, low grade (C54.1)](http://codes.iarc.fr/code/3456) |
| 89353 | Stromal sarcoma, NOS |
| 90453 | Biphenotypic sinonasal sarcoma |

**Table J**. List of ICD-O-3 codes according to molecular alterations.

| **Genomic Alterations** | **List of morphology terms** |
| --- | --- |
| **Complex genomic alterations** | Undifferentiated pleomorphic sarcoma (88023) |
|  | Adult fibrosarcoma (88103) |
|  | Myxofibrosarcoma (88113) |
|  | Periosteal fibrosarcoma (88123) |
|  | Fascial fibrosarcoma (88133) |
|  | Undifferentiated high-grade pleomorphic sarcoma of bone (88303) |
|  | Low-grade fibromyxoid sarcoma (88403) |
|  | Pleomorphic liposarcoma (88543) |
|  | Leiomyosarcoma (excluding skin) (88903) |
|  | Pleomorphic rhabdomyosarcoma (89013) |
|  | Ectomesenchymoma (89213) |
|  | Angiosarcoma of soft tissue (91203) |
|  | Conventional osteosarcoma (91803) |
|  | Chondroblastic osteosarcoma (91813) |
|  | Fibroblastic osteosarcoma (91823) |
|  | Telangiectatic osteosarcoma (91833) |
|  | Secondary osteosarcoma (91843) |
|  | Small cell osteosarcoma (91853) |
|  | Periosteal osteosarcoma (91933) |
|  | High-grade surface osteosarcoma (91943) |
|  | Intracortical osteosarcoma (C40._,C41._) (91953) |
|  | Chondrosarcoma grade II, grade III (92203) |
|  | Chondroblastoma (/3 with metastasis) (92301) |
|  | Clear cell chondrosarcoma (92423) |
|  | Dedifferentiated chondrosarcoma (92433) |
|  | Malignant peripheral nerve sheath tumor (95403) |
|  | Epithelioid malignant nerve sheath tumor (95423) |
|  | Malignant Triton tumor (95613) |
|  | Malignant perineurioma (95713) |
|  | Malignant granular cell tumor (95803) |
| ***MDM2* amplification** | Atypical lipomatous tumor (88501) |
|  | Dedifferentiated liposarcoma (88583) |
|  | Intimal sarcoma (91373) |
|  | Low-grade central osteosarcoma (91873) |
|  | Parosteal osteosarcoma (91923) |
|  | Well differentiated liposarcoma /Dedifferentiated liposarcoma (88501 /88583) |
| **Mutations** | Epithelioid sarcoma (88043) |
|  | Spindle cell / sclerosing rhabdomyosarcoma (89123) |
|  | Gastrointestinal stromal tumor, malignant (89363) |
|  | Extra-renal rhabdoid tumor (89633) |
|  | Atypical teratoïd/rhabdoïd tumor (95083) |

| **Genomic Alterations** | **List of morphology terms** |
| --- | --- |
| **Recurrent translocations** | Desmoplastic small round cell tumor (88063) |
|  | Infantile fibrosarcoma (88143) |
|  | Solitary fibrous tumor, malignant (88153) |
|  | Dermatofibrosarcoma protuberans (88321) |
|  | Pigmented dermatofibrosarcoma protuberans (88331) |
|  | Fibrosarcomatous dermatofibrosarcoma protuberans (88333) |
|  | Ossifying fibromyxoid tumor, malignant (88423) |
|  | Round cell \ Myxoid liposarcoma (88523) |
|  | Alveolar rhabdomyosarcoma (incl. solid, anaplastic) (89203) |
|  | Endometrial stromal sarcoma, NOS (C54.1) (89303) |
|  | Endometrial stromal sarcoma, low grade (C54.1) (89313) |
|  | Stromal sarcoma, NOS (89353) |
|  | Mixed tumor NOS, malignant (89403) |
|  | Clear cell sarcoma of kidney (C64.9) (89643) |
|  | Myoepithelial carcinoma (89823) |
|  | Phosphaturic mesenchymal tumor, malignant (89903) |
|  | Synovial sarcoma NOS (90403) |
|  | Synovial sarcoma, spindle cell (90413) |
|  | Synovial sarcoma, epithelioid cell (90423) |
|  | Synovial sarcoma, biphasic (90433) |
|  | Clear cell sarcoma of soft tissue (90443) |
|  | Biphenotypic sinonasal sarcoma (90453) |
|  | Epithelioid haemangioendothelioma (91333) |
|  | Extraskeletal myxoid chondrosarcoma (92313) |
|  | Mesenchymal chondrosarcoma (92403) |
|  | Extraskeletal Ewing sarcoma (93643) |
|  | Alveolar soft-part sarcoma (95813) |
| **Undefined/Miscellaneous alterations** | Malignant glomus tumor (87113) |
|  | PEComa NOS, malignant (87143) |
|  | Undifferentiated / Unclassified Sarcomas (88003) |
|  | Undifferentiated spindle cell sarcoma (88013) |
|  | Undifferentiated round cell sarcoma (88033) |
|  | Undifferentiated sarcoma NOS (88053) |
|  | Low-grade myofibroblastic sarcoma (88253) |
|  | Liposarcoma, not otherwise specified (88503) |
|  | Rhabdomyosarcoma, SAI (89003) |
|  | Embryonal rhabdomyosarcoma (incl. botryoid, anaplastic) (89103) |
|  | Embryonal sarcoma (89913) |
|  | Kaposi sarcoma (91403) |
|  | Malignancy in giant cell tumor of bone (92503) |
|  | Giant cell tumor of soft parts, NOS (92513) |
|  | Tenosynovial giant cell tumor malignant (92523) |
|  | Adamantinoma (92613) |
|  | Chordoma, NOS (93703) |
|  | Chondroïd chordoma (93713) |
|  | Dedifferentiated chordoma (93723) |

**Table J (continuation)**. List of ICD-O-3 codes according to molecular alterations.
