# Supplementary figures and images for: Incidence and time trends of sarcoma (2000–2013): results from the French network of cancer registries (FRANCIM)
Source: BMC Cancer. 2020 Mar 6;20:190. doi: 10.1186/s12885-020-6683-0 (PMC7059296; doi:10.1186/s12885-020-6683-0)

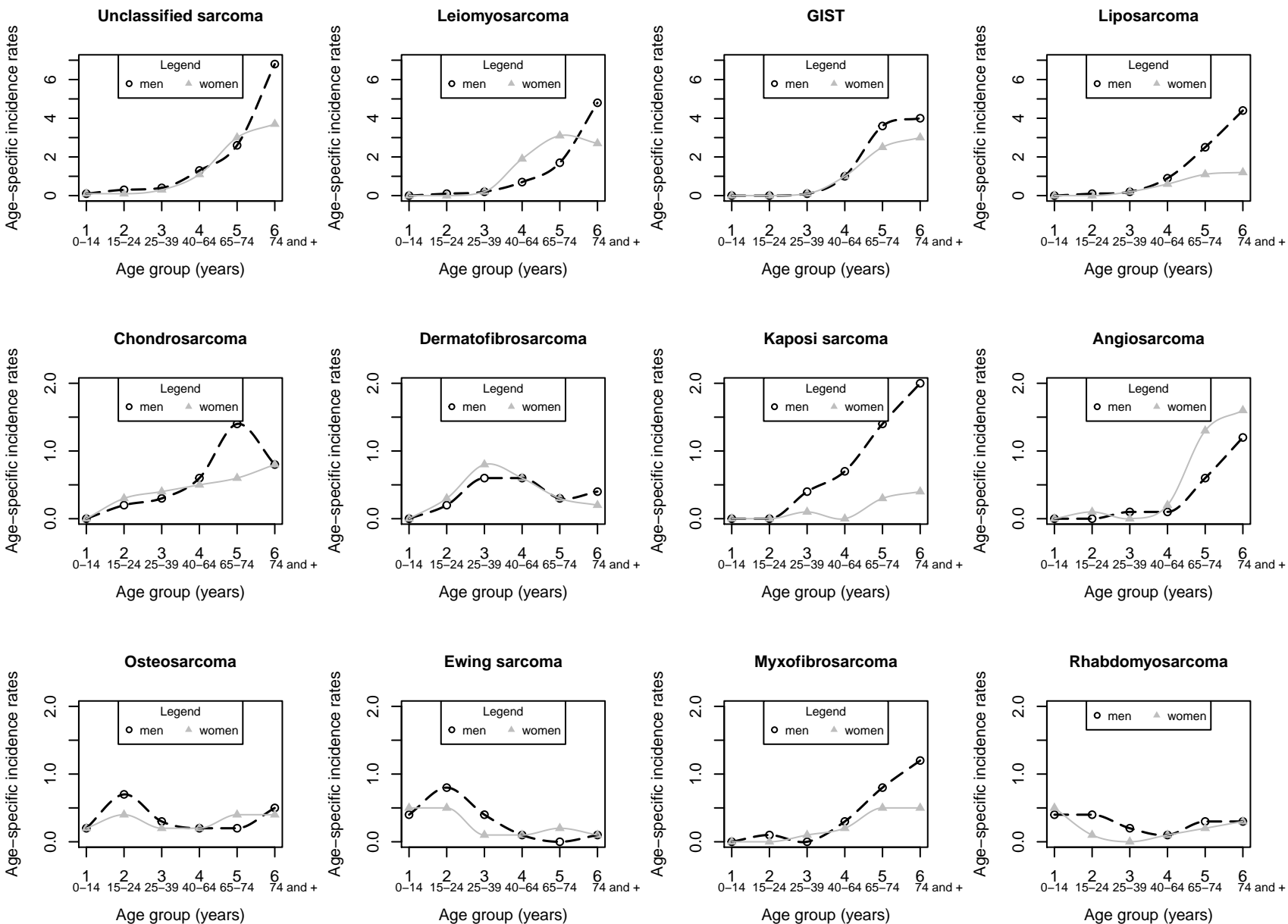

Supplement: Supplementary file 2 — Additional file 2: Figure S1. Age-specific incidence rates of sarcomas per 100,000 person-years according to histologic groups. FRANCIM network data 2010–2013 (19 registries). [file 12885_2020_6683_MOESM2_ESM.pdf]

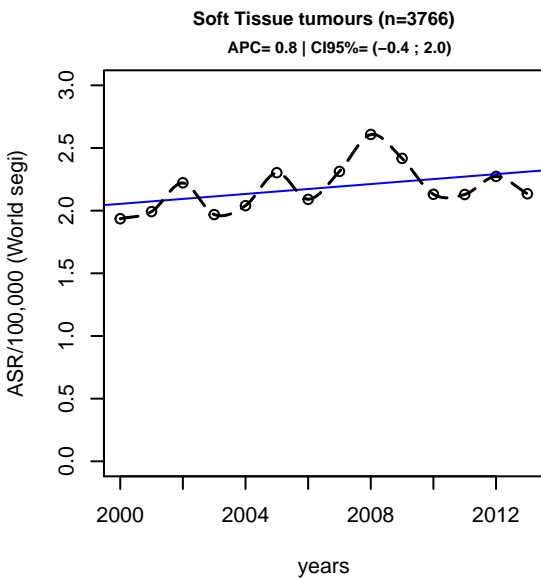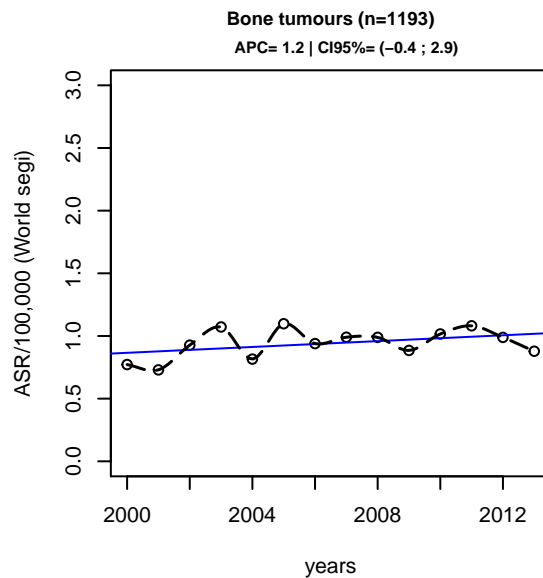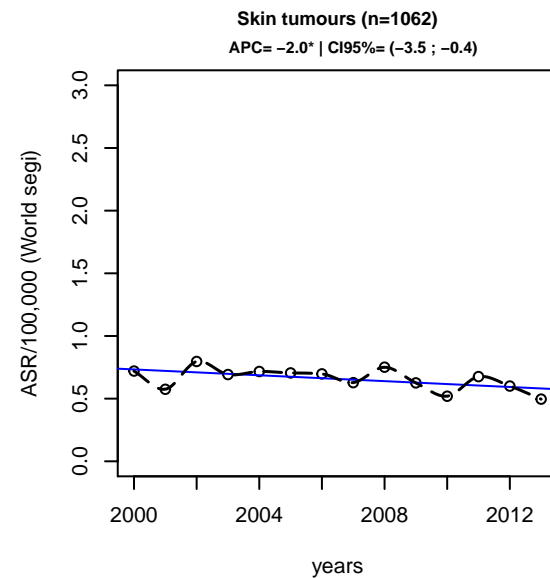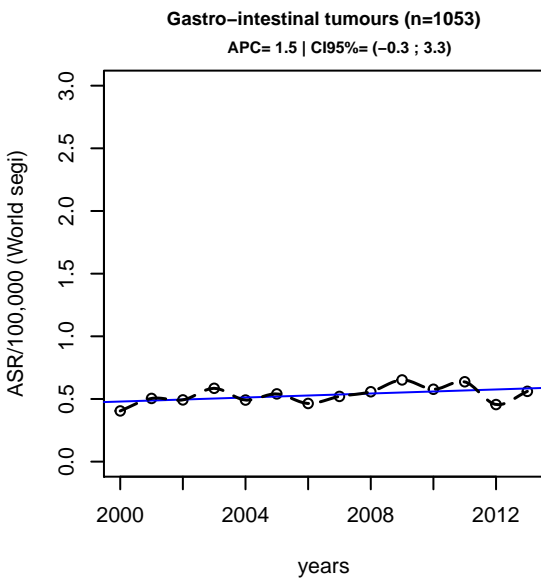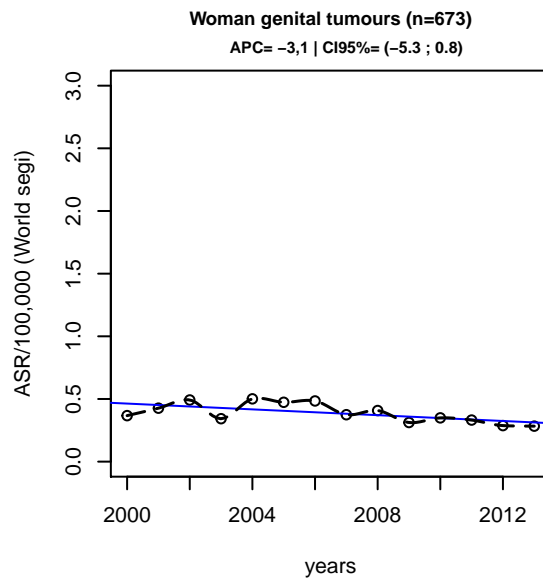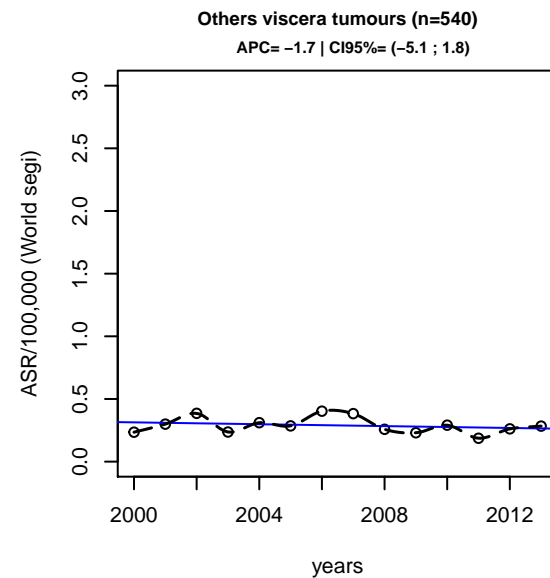

Supplement: Supplementary file 4 — Additional file 4: Figure S3. Sarcoma trends and annual percentage change (APC) of world age-standardized incidence rate according to topographic group. FRANCIM network data 2000–2013 (11 registries). [file 12885_2020_6683_MOESM4_ESM.pdf]
