# Supplementary material for: Incidence and time trends of sarcoma (2000–2013): results from the French network of cancer registries (FRANCIM)
Source: BMC Cancer. 2020 Mar 6;20:190. doi: 10.1186/s12885-020-6683-0 (PMC7059296; doi:10.1186/s12885-020-6683-0)
Supplement: Supplementary file 3 — Additional file 3: Figure S2. Age-specific incidence rates of sarcomas per 100,000 person-years according to genomic groups. FRANCIM network data 2010–2013 (19 registries). [file 12885_2020_6683_MOESM3_ESM.pdf]

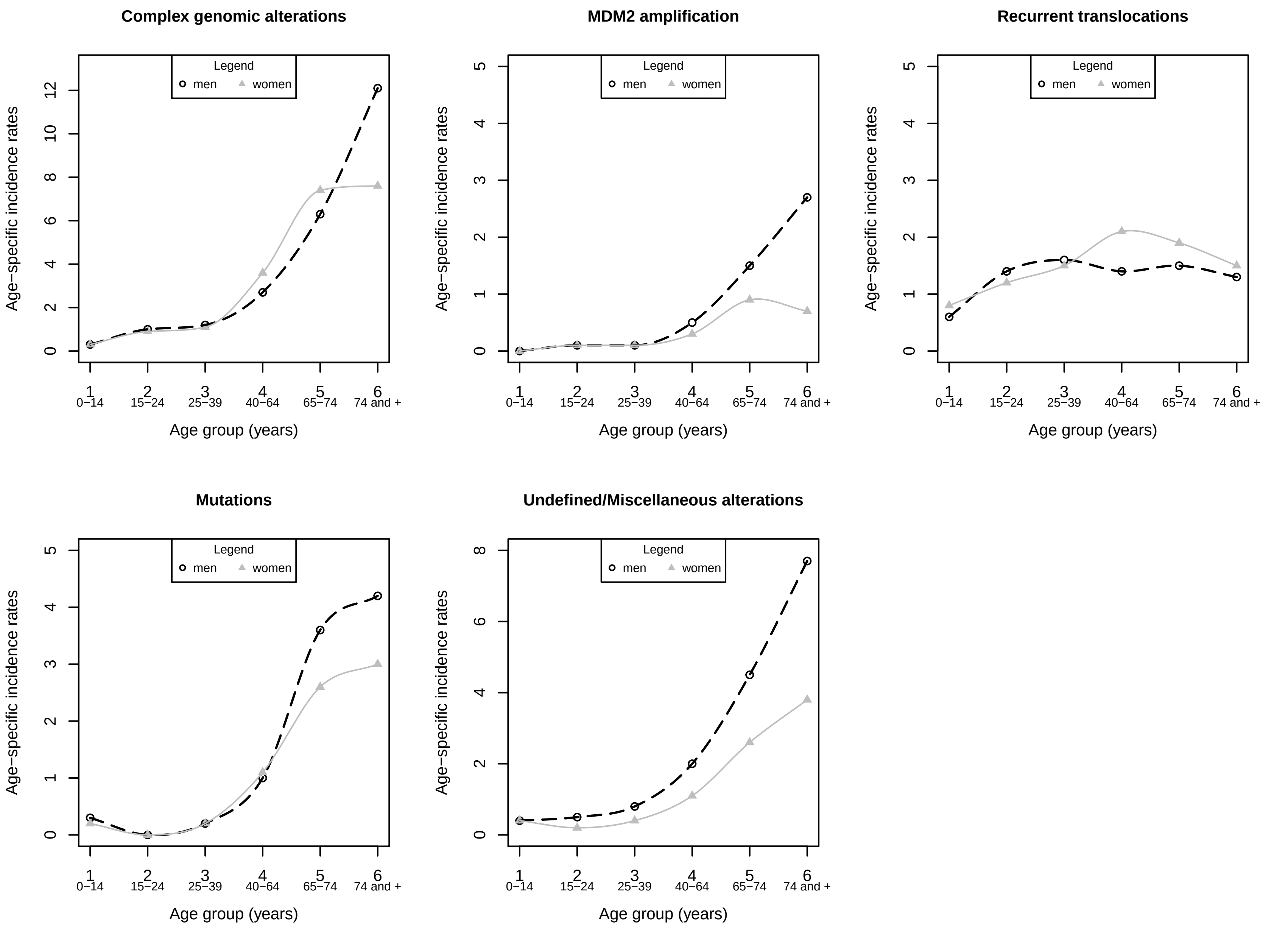

Complex genomic alterations
MDM2 amplification
Recurrent translocations
Mutations
Undefined/Miscellaneous alterations
Legend
men
women
Age−specific incidence rates
Age group (years)
1 0−14
2 15−24
3 25−39
4 40−64
5 65−74
6 74 and +
