# Supplementary material for: Incidence and time trends of sarcoma (2000–2013): results from the French network of cancer registries (FRANCIM)
Source: BMC Cancer. 2020 Mar 6;20:190. doi: 10.1186/s12885-020-6683-0 (PMC7059296; doi:10.1186/s12885-020-6683-0)
Supplement: Supplementary file 5 — Additional file 5: Figure S4. Sarcoma trends and annual percentage change (APC) of world age-standardized incidence rate according to histologic group. FRANCIM network data 2000–2013 (11 registries). [file 12885_2020_6683_MOESM5_ESM.pdf]

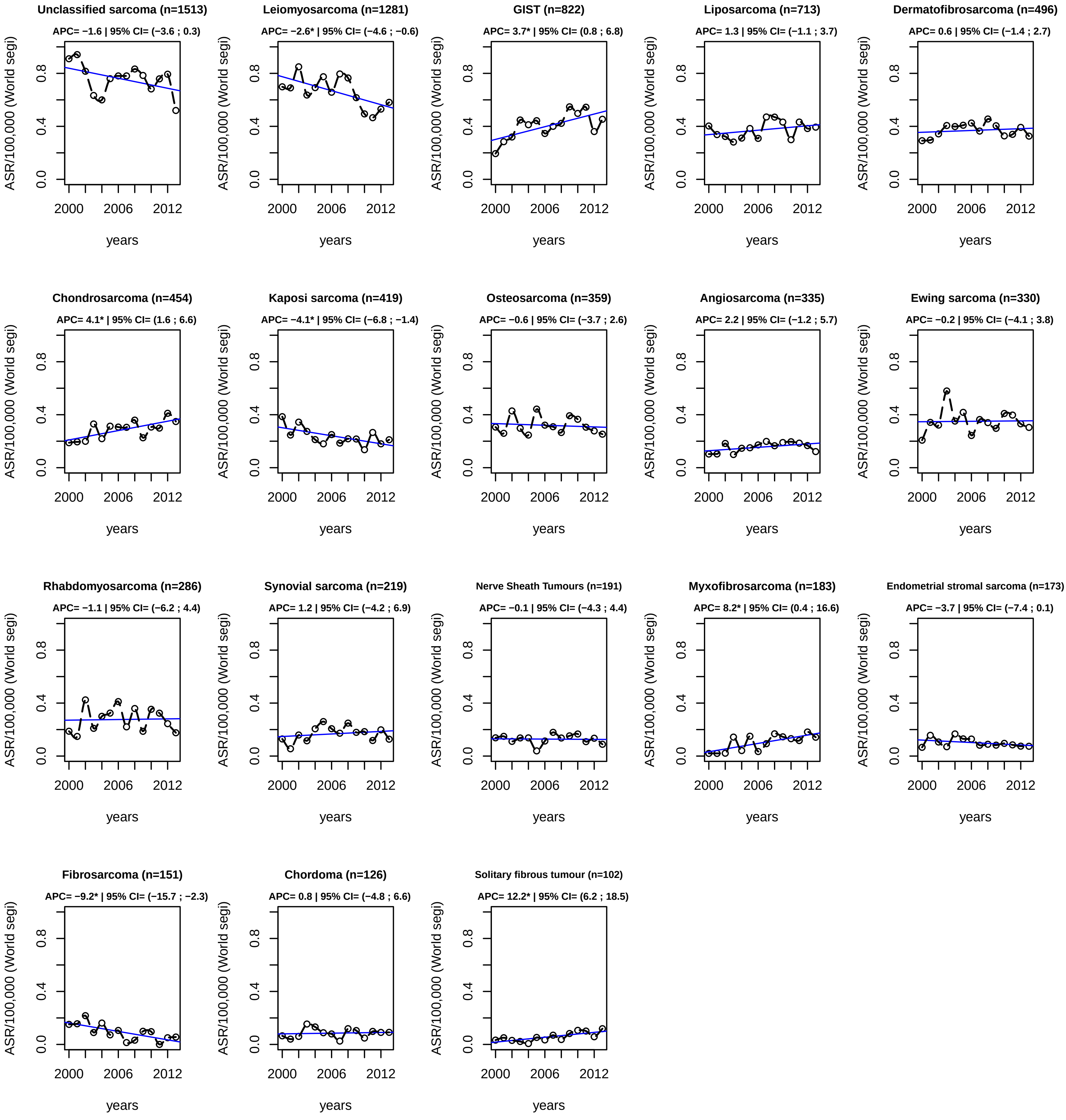
Unclassified sarcoma (n=1513)
APC= −1.6 | 95% CI= (−3.6 ; 0.3)
Leiomyosarcoma (n=1281)
APC= −2.6* | 95% CI= (−4.6 ; −0.6)
GIST (n=822)
APC= 3.7* | 95% CI= (0.8 ; 6.8)
Liposarcoma (n=713)
APC= 1.3 | 95% CI= (−1.1 ; 3.7)
Dermatofibrosarcoma (n=496)
APC= 0.6 | 95% CI= (−1.4 ; 2.7)
Chondrosarcoma (n=454)
APC= 4.1* | 95% CI= (1.6 ; 6.6)
Kaposi sarcoma (n=419)
APC= −4.1* | 95% CI= (−6.8 ; −1.4)
Osteosarcoma (n=359)
APC= −0.6 | 95% CI= (−3.7 ; 2.6)
Angiosarcoma (n=335)
APC= 2.2 | 95% CI= (−1.2 ; 5.7)
Ewing sarcoma (n=330)
APC= −0.2 | 95% CI= (−4.1 ; 3.8)
Rhabdomyosarcoma (n=286)
APC= −1.1 | 95% CI= (−6.2 ; 4.4)
Synovial sarcoma (n=219)
APC= 1.2 | 95% CI= (−4.2 ; 6.9)
Nerve Sheath Tumours (n=191)
APC= −0.1 | 95% CI= (−4.3 ; 4.4)
Myxofibrosarcoma (n=183)
APC= 8.2* | 95% CI= (0.4 ; 16.6)
Endometrial stromal sarcoma (n=173)
APC= −3.7 | 95% CI= (−7.4 ; 0.1)
Fibrosarcoma (n=151)
APC= −9.2* | 95% CI= (−15.7 ; −2.3)
Chordoma (n=126)
APC= 0.8 | 95% CI= (−4.8 ; 6.6)
Solitary fibrous tumour (n=102)
APC= 12.2* | 95% CI= (6.2 ; 18.5)
ASR/100,000 (World segi)
years
0.0
0.4
0.8
2000
2006
2012
